# Prevalence and associated factors of skin cancer in aged nursing home residents: A multicenter prevalence study

**DOI:** 10.1371/journal.pone.0215379

**Published:** 2019-04-22

**Authors:** Merve Akdeniz, Elisabeth Hahnel, Claas Ulrich, Ulrike Blume-Peytavi, Jan Kottner

**Affiliations:** 1 Department of Dermatology and Allergy, Clinical Research Center for Hair and Skin Science, Charité-Universitätsmedizin Berlin, Germany; 2 Department of Dermatology and Allergy, Skin Cancer Center, Charité-Universitätsmedizin, Berlin, Germany; University Magna Graecia of Catanzaro, ITALY

## Abstract

Non-melanoma-skin cancer is an emerging clinical problem in the elderly, fair skinned population which predominantly affects patients aged older than 70 years. Its steady increase in incidence rates and morbidity is paralleled by related medical costs. Despite the fact that many elderly patients are in need of care and are living in nursing homes, specific data on the prevalence of skin cancer in home care and the institutional long-term care setting is currently lacking. A representative multicenter prevalence study was conducted in a random sample of ten institutional long-term care facilities in the federal state of Berlin, Germany. In total, n = 223 residents were included. Actinic keratoses, the precursor lesions of invasive cutaneous squamous cell carcinoma were the most common epithelial skin lesions (21.1%, 95% CI 16.2 to 26.9). Non-melanoma skin cancer was diagnosed in 16 residents (7.2%, 95% CI 4.5 to 11.3). None of the residents had a malignant melanoma. Only few bivariate associations were detected between non-melanoma skin cancer and demographic, biographic and functional characteristics. Male sex was significantly associated with actinic keratosis whereas female sex was associated with non-melanoma skin cancer. Smoking was associated with an increased occurrence of non-melanoma skin cancer. Regular dermatology check-ups in nursing homes would be needed but already now due to financial limitations, lack of time in daily clinical practice and limited number of practising dermatologists, it is not the current standard. With respect to the worldwide growing aging population new programs and decisions are required. Overall, primary health care professionals should play a more active role in early diagnosis of skin cancer in nursing home residents. Dermoscopy courses, web-based or smartphone-based applications and teledermatology may support health care professionals to provide elderly nursing home residents an early diagnosis of skin cancer.

## Introduction

The growing and aging of the world population [1, 2] is associated with an increase of aging-related skin conditions and cutaneous diseases [3]. One of the most distressing and especially in elderly patients fatal skin disease, is skin cancer [4]. There are two types of primary skin cancer, developing within the epidermis: melanoma and non-melanoma skin cancer (NMSC) [5]. Basal cell carcinoma (BCC) and cutaneous squamous cell carcinoma (cSCC) are referred to NMSC. In addition, actinic keratosis (AK), also known as solar keratosis is characterized by atypical epidermal keratinocytes, represents a preinvasive “in-situ” lesion of SCC [6].

The incidence of both NMSC and melanoma skin cancers has been increasing [7]. The highest incidence rates have been reported in New Zealand with 50 cases per 100,000 people and Australia with 48 cases per 100,000 people (59 for males and 39 for females in 2011), followed by the US (21.6 new cases yearly per 100,000 in 2012) and Europe (13.2 and 13.1 new cases yearly, per 100,000 for men and women, respectively). The incidence of NMSC is 18–29 times higher than melanoma [8]. Each year, between two and three million NMSC occur globally and nearly 50% of the U.S. population will have skin cancer at least once until the age of 65 years [7, 9].

Beside genetic susceptibility and sufficient immunological surveillance, environmental factors, mainly chronic exposure to ultraviolet (UV) radiation, ionizing radiation, and chemical carcinogens such as arsenic exposure are acknowledged as important risk factors for NMSC [10–14]. Smoking has been reported as an additional, independent risk factor for NMSC by some authors [15]. However, results of these studies are conflicting, reporting both positive and negative associations [16]. Empirical evidence also shows a strong association between androgenetic alopecia and NMSC [17].

Latest epidemiological figures indicate that the prevalence of skin cancer is 0.6% to 13.5% in patients of acute or chronic geriatric units or hospitals [4, 18–20]. According to Nursing Care Statistics of Germany 2015, about 800.000 residents receive care in long-term care institutions. However, the skin cancer prevalence in institutional long-term care facilities is mostly unknown [20]. Representing a fragile and increasing group, a detailed knowledge about the load of skin cancer in this sub-population is extremely relevant, because the regular provision of dermatological examination in these care settings is not common [21]. Results of a recent survey conducted in the Netherlands revealed that only 30% of the interviewed dermatologists and dermatology residents ever visited a patient within a nursing home. However, the most common reasons for dermatological visits were cutaneous (pre)malignancies (51.4%) [22]. The knowledge of frequency and prevalence in given age groups may help to develop a screening schedule for these long-term care residents, showing a high skin cancer risk. Thus, the aim of the current study was to measure the prevalence of precursor lesions and skin cancer in residents living in institutional long-term care facilities and to explore possible associations with demographic, biographic and functional characteristics.

## Methods

### Study design and setting

An observational, cross-sectional prevalence study was conducted from September 2014 to May 2015 in ten institutional long-term care facilities in Berlin, Germany. A study protocol was published before (https://clinicaltrials.gov/ct2/show/NCT02216526) and was approved by the ethics committee of Charité-Universitätsmedizin Berlin (EA1/190/14). A detailed description of the study procedures has been published [23]. Institutional long-term care facilities from a list of all existing facilities (n = 291 in 2014) in the federal state of Berlin were contacted in a random order and invited to participate.

### Participants

Participants had to meet the following inclusion criteria: (1) being resident of the respective nursing home facility, (2) being 65+ years, (3) written informed consent (or by legal representative). Residents at the end of life were excluded. All residents living in the nursing home facility at the time of data collection were invited to participate by study assistants and investigators. Written informed consent was obtained from the residents or their legally authorized representatives on their behalf.

### Variables

Skin malignancies, pre-malignancies, androgenetic alopecia and melanocytic nevi were classified according to the International Classification of Diseases (ICD-10). Demographics (age, sex and BMI), biographic (qualification and occupation), physical and functional (Barthel Index) characteristics were measured. The Barthel Index (BI) was used to assess the dependency in the activities of daily living [24]. The educational level was classified into six categories: (1) no school qualification, (2) primary school, (3) secondary school, (4) grammar school/A-level, (5) vocational training and (6) university qualification. The smoking status was classified into three categories: (1) smoker, (2) former smoker, (3) never smoked. The history of employment was classified into two categories: (1) indoor, (2) outdoor.

### Data sources and measurement

The clinical dermatological examination was performed by board certified dermatologists for each participating nursing home resident. Clinical examinations and evaluations were performed with dermatoscopes (Dermogenius basic, DermoScan GmbH, Germany). Demographic, biographic and functional characteristics were obtained from the medical records or assessed by a study assistant via interview of the responsible nurse or the resident were interviewed if possible (depending on the cognitive abilities).

### Bias

In order to reduce selection bias, all nursing homes of the federal state of Berlin were randomly selected. Measurements and clinical procedures were performed by trained board certified dermatologists and study assistants according to standard operating procedures. To reduce the risk of detection bias, the dermatologists had no access to medical history of the residents prior and during examinations.

### Study size

Assuming a prevalence of 0.5 of skin diseases, we regarded approximately n = 280 residents as sufficient to measure this proportion with a desired width of a 95% CI of ± 0.06. According to the Nursing Care Statistics (2013), the size of the long-term care population in Berlin was approximately n = 30.000. Assuming n = 80 residents per institution and a participation rate of 50% (n = 40), it was planned to include seven institutions which results in n = 280 (7 x n = 40) cases.

### Quantitative variables

The BI was used as metric variable. In order to detect possible associations with skin diseases, the variable educational qualification, smoking status and the outdoor occupation were dichotomized into university qualification (yes/no), smoking (ever/never) and outdoor occupation (yes/no). Outdoor occupation was evaluated based on self-reported occupational histories from the data collection forms by two reviewers independently (MA, EH). Any uncertainties were discussed and resolved by a third reviewer (JK).

### Statistical methods

Depending on the level of measurement (nominal, ordinal and continuous), demographic characteristics, functional assessment scores and dermatological diseases were described using means, medians proportions, frequencies and associated spread estimates such as standard deviations and (interquartile) ranges. The 95% CIs were calculated around point estimates of dermatological diseases. Exploratory data analysis to investigate possible bivariate associations were conducted using logistic regression analysis for skin diseases and odds ratios (OR) were calculated. 95% CIs of the ORs excluding 1 were considered to be statistically significant. ORs being statistically significant or with values lower than 0.5 or higher than 2.0 were considered to be likely associated.

## Results

### Participants

In order to achieve the planned number of participants, three additional long-term care facilities were recruited. In total, 10 from 55 contacted long-term care facilities agreed to participate. In total n = 811 long-term care residents were living within the nursing homes at time of data collection and therefore potentially suitable for participation. N = 252 residents provided written informed consent by themselves or by their legal representative. Twenty-nine residents declined participation prior examination resulting in n = 223 included long-term care residents.

### Descriptive data and main results

Sample characteristics are shown in Table 1. Most residents were female (67.7%) and the mean age was 83.6 (SD 8.0) years. Mean BMI was 25.3 (SD 5.1) kg/m^2^. Mean BI was 45.1 (SD 23.8). A vocational training was the highest educational level for the majority (48.9%). Seventeen residents (8.5%) had an outdoor work history. Fifty-two residents (51.0%) have never smoked, whereby 37 residents (36.3%) were former and 13 residents (12.7) were current smoker. Androgenetic alopecia was diagnosed in n = 112 residents (50.2%). Twenty-nine residents (13.0%) were diagnosed with melanocytic nevi. The most common pre-cancerous skin lesion was actinic keratosis, the precursor lesions of cSCC (n = 47; 21.1%). Sixteen residents (7.2%) were diagnosed with NMSC. BCC was detected in 15 residents (6.7%) and was the most common NMSC. Only one resident (0.4%) was diagnosed with cSCC. Bowen’s disease (SCC in situ) was diagnosed in seven residents (3.1%). One resident (0.4%) was diagnosed with lentigo maligna. None of the residents had malignant melanoma.

**Table 1:** Characteristics of nursing home residents (n = 223).

**Female, n (%)**	151 (67.7)
**Age (years)**	
Mean (SD)	83.6 (8.0)
Median (IQR)	84 (78–89)
**BMI (kg/m^2^) ^1^**	
Mean	25.3 (5.1)
Median	24.6 (21.9–28.3)
**Barthel Index total score^2^**	
Mean (SD)	45.1 (23.8)
Median (IQR)	45.0 (25.0–65.0)
**Outdoor occupation, n (%)**	17/200 (8.5)
**Smoking status**	
Non-smoker, n (%)	52/102 (51.0)
Smoker, n (%)	13/102 (12.7)
Former smoker, n (%)	37/102 (36.3)
**Highest educational qualification, n (%)**	
No school qualification	3/184 (1.6)
Primary school	34/184 (18.5)
Secondary school	24/184(13.0)
Grammar school/A-level	7/184 (3.8)
Vocational Training	90/184 (48.9)
University	26/184 (14.1)
**Androgenetic alopecia (L64.9)**	112 (50.2%) (95% CI 43.7–56.7)
**Melanocytic Nevi (D22.9)**	29 (13.0%) (95% CI 9.2–18.1)
**Actinic keratosis, n (%) (L57.0)**	47 (21.1) (95% CI 16.2–26.9)
Cheilitis actinica, n (%) (L56.8)	10 (4.5)
Actinic keratosis head	29 (13.0)
Actinic keratosis trunk	2 (0.9)
Actinic keratosis arms	6 (2.7)
Actinic keratosis, hands	1 (0.4)
Actinic keratosis, legs	1 (0.4)
**Bowen´s Disease, n (%) (D04.9)**	7 (3.1) (95% CI 1.5–6.3)
**Lentigo Maligna, n (%) (D03.9)**	1 (0.4) (95% CI 0.1–2.5)
**Non-melanoma skin cancer, n (%) (C44.9)**	16 (7.2) (95% CI 4.5–11.3)
Basal cell carcinoma, n (%)	15 (6.7)
Basal cell carcinoma head, n (%)	11 (4.9)
Basal cell carcinoma trunk	4 (1.8)
Basal cell carcinoma arms	1 (0.4)
Cutaneous squamous cell carcinoma, n (%)	1 (0.4) (95% CI 0.1–2.5)
**Malignant melanoma, n (%) (C43.9)**	0 (0)

^1^ n,216

^2^ n,222

The results of the bivariate associations between NMSC, actinic keratosis, androgenetic alopecia, melanocytic nevi and demographic, biographic and functional characteristics are shown in Table 2. Female sex showed a significant negative association with the presence of actinic keratosis (OR 0.321, 95% CI 0.165 to 0.622) and an increased occurrence of NMSC (OR 2.167, 95% CI 0.597 to 7.857). Female sex was also associated with a decreased occurrence of androgenetic alopecia (OR 0.187, 95% CI 0.099 to 0.354). Age and care dependency (BI) seem not to be associated with non-melanoma skin cancer. Having an outdoor occupation history was associated with an increased occurrence of melanocytic nevi (OR 2.140, 95% CI 0.643 to 7.127). Having a university qualification was associated with an increased occurrence of Bowen´s disease (OR 3.200, 95% CI 0.588 to 17.409). Smoking or being a former smoker was associated with an increased occurrence of NMSC (OR 2.223, 95% CI 0.766 to 6.451).

**Table 2:** Associations between skin diseases and demographic characteristics (bivariate).

Skin diseases (ICD-10)	Age (years) (OR, 95% CI)	Sex (OR, 95% CI) (0 = male, 1 = female)	Barthel Index (OR, 95% CI)	Outdoor occupation (0 = no, 1 = yes) (OR, 95% CI)	University Qualification (0 = no, 1 = yes) (OR, 95% CI)	Smoking Status (0 = never, 1 = ever) (OR, 95% CI)
Actinic keratosis (L57.0)	1.029 (0.988 to 1.071)	**0.321** (0.165 to 0.622)	1.004 (0.991 to 1.018)	1.100 (0.340 to 3.559)	1.141 (0.431 to 3.026)	0.919 (0.420 to 2.010)
Bowen’s Disease (D04.9)	1.104 (0.994 to 1.226)	0.626 (0.136 to 2.873)	0.996 (0.965 to 1.028)	No results	3.200 (0.588 to 17.409)	0.568 (0.067 to 4.832)
Non-melanoma skin cancer (C44.9)	1.061 (0.992 to 1.134)	2.167 (0.597 to 7.857)	1.002 (0.981 to 1.024)	0.700 (0.087 to 5.649)	1.089 (0.233 to 5.089)	2.223 (0.766 to 6.451)
Melanocytic Nevi (D22.9)	0.955 (0.908 to 1.004)	0.634 (0.285 to 1.411)	1.008 (0.992 to 1.025)	2.140 (0.643 to 7.127)	1.251 (0.398 to 3.931)	0.690 (0.249 to 1.912)
Androgenetic alopecia (L64.9)	0.984 (0.952 to 1.017)	0.187 (0.099 to 0.354)	0.999 (0.988 to 1.010)	1.979 (0.702 to 5.578)	1.179 (0.519 to 2.676)	1.096 (0.584 to 2.057)

Underlined values indicate OR ≥ 2.0 or OR ≤ 0.5, bold values indicate statistical significance

Results of the bivariate associations between non-melanoma skin cancer, actinic keratosis, Bowen’s disease, androgenetic alopecia and melanocytic nevi are shown in Table 3. An increased occurrence of Bowen´s disease was associated with an increased occurrence of non-melanoma skin cancer (OR 2.233, 95% CI 0.252 to 19.777). There was also an association between the increased occurrence of melanocytic nevi and increased occurrence of actinic keratosis (OR 2.682, 95% CI 1.166 to 6.169).

**Table 3:** Associations between skin diseases (bivariate).

Skin diseases (ICD-10)	Actinic keratosis (L57.0)	Bowen Disease (D04.9)	Non-melanoma skin cancer (C44.9)	Androgenetic alopecia (L64.9)	Melanocytic Naevi (D22)
Actinic keratosis (L57.0)	.				
Bowen’s Disease (D04.9)	1.520 (0.285 to 8.094)	.			
Non-melanoma skin cancer (C44.9)	1.271 (0.391 to 4.139)	2.233 (0.252 to 19.777)	.		
Androgenetic alopecia (L64.9)	1.445 (0.755 to 2.767)	0.736 (0.161 to 3.368)	0.572 (0.200 to 1.631)	.	
Melanocytic Naevi (D22.9)	2.682 (1.166 to 6.169)	No results	0.952 (0.205 to 4.424)	No results	.

Underlined values indicate OR ≥2.0; OR ≤0.5

## Discussion

### Key results

Nearly every fifth nursing home resident was affected by actinic keratosis. 7.2% were affected by non-melanoma skin cancer, which shows a high load of skin malignancies in this population. Actinic keratosis was most strongly associated with male sex. NMSC was most strongly associated with smoking and female sex.

### Limitations

The exclusion of residents at the end of life and the lower participation rate than expected of n = 223/811 residents may have caused a selection bias. It is well known that UV-exposure plays an important role in the development of AK and NMSC but the intensity and the duration of UV exposure was not measured. Furthermore, other indicators for cumulative UV exposure such as outdoor hobbies, sunburns in the childhood or sun protection habits were not assessed. Likewise, we have not evaluated the intensity and the duration of the smoking history, which might be another important modifiable risk factor.

### Interpretation

Our results indicate that every fifth aged nursing home resident was affected by AK. This was the most commonly detected precancerous lesion and the prevalence is similar to previous reports [20, 25]. According to our findings, male sex is associated with the occurrence of actinic keratosis that is most probably because of the increased prevalence of alopecia. A weak positive association between AK and alopecia could be shown in our data as well. Chronic sun exposure is a major risk factor for the development of these lesions and the usual detection of AKs in frequently sun-exposed areas (e.g. balding scalp, face, distal upper extremities). Expectedly, AKs were detected on frequently sun-exposed areas; head and arms. In a Dutch population-based cohort study of n = 2061 men and women over the age of 50, severe baldness was associated with increased risk of AK when compared with minimal or no baldness, whereas outdoor work history and educational level showed no statistical association with the occurrence of AK [26], which supports our findings.

Our findings support previous studies, indicating sex influences the risk of AK [27, 28]. In a study conducted in Queensland, Australia [28], AK lesions were detected in 79% of men and 68% of women between the ages of 60 and 69 compared with only 10% of men and 5% of women aged 20 to 29 years in n = 2045 adults between the ages of 20 and 69 years. The prevalence of AK was much higher in this younger population than our study findings, which is most probably resulting from the geographic location.

Our results indicate a positive association between the increased occurrence of actinic keratosis and melanocytic nevi. A previous study examining risk factors for actinic keratosis in eight European cities reported that the overall number of benign nevi to be inversely associated with AK, yet no significant association with the presence of benign nevi [29]. An explanation may be that we did not compare the numbers of nevi but the presence of nevi with the presence of AK.

It is well known that UV-exposure habits and older age play important roles in the development of AK and NMSC. Interestingly, our results did not indicate an association between outdoor occupation and the occurrence of AK or NMSC. This might be due to the lack of data regarding the intensity and the duration of the outdoor working activity. In fact there was a positive relationship between AK and alopecia as well (1.445%95 CI 0.755 to 2.767) but compared to other variables this association was weak. Additionally, our study population had a high mean age of 83.6 years. Aging and cancer is a known biological phenomenon. However, several earlier studies also reported that immune senescence might paradoxically reduce tumor growth rate with aging [30, 31]. The same mechanism might also apply for NMSC.

One of 32 nursing home residents were affected by NMSC. The estimated prevalence is similar to previous reports [4]. Although a previous study examining the association between male pattern baldness at age of 45 and the risk of incident cSCC and BCC, indicates a significant association between increased risk of NMSC and androgenetic alopecia [17], our results demonstrates a lack of association between androgenetic alopecia and non-melanoma skin cancer. In contrast to previous epidemiological observations, our findings also indicated a higher risk for female residents to develop NMSC compared to males [32].

Furthermore, it has been reported that the risk for developing cSCC is higher among people who are current or former smoker [33]. On the other hand, it is unclear whether smoking increases the risk for BCC. Several studies suggest an association between smoking and the occurrence of BCC, whereas some studies have shown no relationship [16, 34]. Our results indicate a positive association between smoking and the overall risk of NMSC.

The associations between Bowen’s disease and NMSC were expected. Etiological factors for Bowen’s disease and NMSC all include chronic exposure to UV radiation in sunlight. Still, it might be an incidental finding, which may have led to a wide confidence interval (OR 2.233 95% CI 0.252 to 19.777). University qualification was also associated with the increased occurrence of Bowen’s disease. To the best of our knowledge, there is no scientific explanation for this. It might be an incidental finding, which may also have led to a wide confidence interval (OR 3.200 95% CI 0.588 to 17.409), both resulting from the small sample size of the residents with a Bowen’s disease (n = 7). This might limit these findings.

According to our findings, the occurrence of melanocytic nevi seems to be related with an outdoor occupation history of the nursing home residents. A previous study examining the relationship between sun exposure and the presence of nevi reported higher nevus density in chronically sun-exposed areas in a cohort of children in Colorado compared to intermittently sun-exposed areas [35]. Our results support these findings. However, there is no evidence that the same association will also apply for geriatric patients.

Lentigo maligna was detected only in one resident whereas none of the residents had a malignant melanoma. However, BCC was detected in 15 residents, whereas cSCC was detected in one resident only. BCC is a common seen NMSC in the elderly with a good prognosis and rare metastasis in the majority of cases and can be managed with both surgical and non-surgical modalities. Depending on the anatomical location, therapeutical challenges may still occur (e.g. ear, periorbital, and nose) [36]. Early detection of BCC, choice of best age- and general condition adapted therapy depending on co-morbidities, frailty of patients, the type of tumor and the localisation is very important [4]. Although cSCC is less common than BCC, unlike to BCC high risk cSCC can spread to regional lymph nodes and then metastasize to distant sites, which makes an early diagnosis highly important. As in BCC, complete surgical excision with histopathological control of excision margins is the gold standard treatment for primary invasive cSCC. Limitations can occur in very large and thick tumours, especially in the elderly population.

An oncogeriatric approach in nursing homes for screening and the assessment of the nursing home residents with NMSC is generally lacking. According to a current survey among dermatologists in the Netherlands, only a minority of dermatologists perform visits within nursing homes and diagnostic procedures are less frequently used as compared to outpatient clinics. Financial limitations, lack of time in clinical practice and insufficient numbers of dermatologists are important challenges. It is very unlikely that regular dermatology visits in nursing homes can be performed for the worldwide growing older population. Future clinical practice guidelines should ideally focus on easy to administer methods and should be adaptable to daily life conditions of dermatological and nursing home care settings. Effective education of primary care physicians using easily reproducible, generalizable training programs, dermoscopy courses for healthcare professionals for skin cancer might increase the early detection of skin cancer [37]. Moreover, web-based and smartphone-based applications can train healthcare professionals to detect skin cancer or precancerous skin lesions at the right time and get the nursing home residents to a dermatologist. Furthermore, suspicious clinical and dermoscopic images can be shared immediately with skin cancer consultants [3]. Nurses can also take a more active role in the early diagnosis of skin cancer by questioning the date of last skin examination, by being capable to detect and triage atypical skin lesions with the help of skin cancer trainings [38]. To focus on modifiable risk factors such as developing smoking prevention strategies and creating sun protective habits in the childhood in countries with high incidence of skin cancer may also be supportive in terms of increasing the skin cancer awareness in the general population and in order to reduce the burden of skin cancer in the future.

### Generalisability

In terms of age, sex and BI, the sample characteristics were comparable to typical geriatric and long-term care populations in Germany [39]. The almost exclusively Caucasian based population and the geographic location, may possibly limit the generalizability of the findings.

## Conclusion

Nearly every fifth nursing home resident was affected by actinic keratosis. Our results indicate an association between male sex and actinic keratosis and an association between female sex and NMSC. Previous smoking seems to be associated with an increased incidence of NMSC. Malignant melanoma does not seem to be a frequently occurring tumor in the elderly and very elderly residents living in the institutional long-term care facilities at this age. However, 7.2% of the residents were affected by NMSC. Allied healthcare professionals should play a more important role to deliver dermatological care and to prevent progression from in situ carcinoma (AK) to squamous cell carcinoma.
