# Correction: Prevalence and associated factors of skin cancer in aged nursing home residents: A multicenter prevalence study

**DOI:** 10.1371/journal.pone.0220488

**Published:** 2019-07-25

**Authors:** Merve Akdeniz, Elisabeth Hahnel, Claas Ulrich, Ulrike Blume-Peytavi, Jan Kottner

The following information is missing from the Funding statement: This study was supported by the German Research Foundation (DFG) and the Open Access Publication Fund of Charité –Universitätsmedizin Berlin.
